# Individual Differences in Accent Imitation

**DOI:** 10.1162/opmi_a_00161

**Published:** 2024-08-31

**Authors:** Emily B. Myers, Hannah E. Olson, Jennifer Scapetis-Tycer

**Affiliations:** Department of SLHS, University of Connecticut; Department of Psychological Sciences, University of Connecticut; Department of Dramatic Arts, University of Connecticut

**Keywords:** accent imitation, vocal mimicry, speech alignment, speech articulation

## Abstract

All talkers show some flexibility in their speech, and the ability to imitate an unfamiliar accent is a skill that shows vast individual differences. Yet the source of these individual differences, in particular whether they originate from perceptual, motor, or social/personality factors, is not yet clear. In the current study, we ask how individual differences in these factors predict individual differences in deliberate accent imitation. Participants imitated three accents, and attempts were rated for accuracy. A set of measures tracking individual differences in perceptual, motor, cognitive, personality, and demographic factors were also acquired. Imitation ability was related to differences in musical perception, vocal articulation, and the personality characteristic of “openness to experience,” and was affected by attitudes towards the imitated talkers. Taken together, results suggest that deliberate accent imitation skill is modulated not only by core perceptual and motor skills, but also by personality and affinity to the talker, suggesting that some aspects of deliberate imitation are a function of domain-general constraints on perceptual-motor systems, while others may be modulated by social context.

## INTRODUCTION

Vocal imitation has a long evolutionary history, and the ability to imitate songs or calls is found in many non-human species (Carouso-Peck et al., 2021; e.g., Fitch, 2005; Iacoboni, 2009). In humans, over the course of infancy, babies learn to mimic the vocal patterns (prosody, syllable structure) that are most dominant in their auditory environment, leading to the suggestion that imitation of speech may be a precursor to language development (Kuhl & Meltzoff, 1996). Notably, imitation of the phonetic qualities of speakers around us can be deliberate (e.g., in the case of an actor doing an impression) or apparently automatic (e.g., triggered by exposure to a speech of a person with a different accent; Delvaux & Soquet, 2007).

Individual variation in deliberate imitation of the vocal characteristics of other talkers is substantial (Adank, Rueschemeyer, & Bekkering, 2013; Reiterer et al., 2011, 2013; Schertz et al., 2023). Better deliberate imitators are more likely to have musical training (Turker et al., 2017), especially vocal training (Christiner & Reiterer, 2013, 2015), pointing to a role in perceptual-motor expertise in explicit imitation. Working memory tends to be higher in better imitators (Christiner & Reiterer, 2015; Reiterer et al., 2011), pointing to potential domain-general contributions towards imitation behavior. We note that in studies of vocal imitation (as in psycholinguistic studies generally), few studies report test-retest reliability checks that would allow us to conclude that the imitation behavior is a trait of the individual, rather than measurement noise. Notably, at least one study (Wade et al., 2021) uses a test-retest design to show that individual differences in the degree of spontaneous imitation of another talker are fairly stable (see also Yu & Zellou, 2019 and Heffner et al., 2022 for a discussion of methodological challenges to assessing individual differences in perception and production). This suggests that there may be real underlying differences in the degree to which a talker is able to adjust their speech to resemble the patterns of another talker. Additionally, we can extrapolate from the real-world experiences of dialect coaches in the performing arts that variation in base accent acquisition skills is also observed in that context. To quote veteran film dialect coach Jill McCullough: “you know very very quickly whether somebody can do this or not” (Meier, 2020). However, the literature on predictors of individual differences in *deliberate* imitation is not as extensive as the literature on *spontaneous* imitation behaviors; theoretical frameworks explaining spontaneous imitation enjoy a rich history. This leads us to appeal to this literature to ask whether the frameworks accounting for spontaneous imitation, and the individual differences that have been found to predict spontaneous imitation, can also be borrowed to account for individual differences in deliberate imitation.

A rich body of literature investigating spontaneous imitation of speech (a term we use to capture phenomena variously termed phonetic alignment, phonetic convergence, or phonetic accommodation) has suggested that speech imitation has an important role to play in social alignment (Giles et al., 1991), and that it may be a driver of linguistic innovation (Nguyen & Delvaux, 2015). In studies of spontaneous imitation, participants show shifts in their production to align with the speech of another talker, without any specific instructions to imitate that talker (Babel, 2010, 2012; Babel et al., 2014; Lewandowski & Jilka, 2019; Pardo et al., 2012, 2013, 2018; Yu et al., 2013). Spontaneous imitation was observed during conversational tasks (Lewandowski & Jilka, 2019; Ostrand & Chodroff, 2021; Pardo et al., 2018), shadowing (Babel, 2012; Clopper & Dossey, 2020; Dufour & Nguyen, 2013), and production after passive exposure (Delvaux & Soquet, 2007; Pardo et al., 2012). Yet these studies also consistently report widespread individual variability in the degree of spontaneous imitation that talkers engage in (Pardo et al., 2018).

Should we expect that predictors of individual differences in spontaneous imitation are also useful for predicting deliberate imitation? The boundaries between spontaneous and deliberate imitation may be fuzzy. Anecdotal reports from expert speech imitators (e.g., impression artists, actors) suggest that excellent deliberate speech imitators may also have a strong pull towards spontaneous speech imitation. Bill Hader, an actor known for accurate vocal impressions, reported in an interview, “… when you’re nervous around someone you start to talk like them … I’ve done this since I was a kid. I think that [experience] lent itself to being able to do voices” (Arnett et al., 2023). One account of spontaneous imitation posits that speech convergence is triggered by automatic internal simulation of speech patterns during comprehension (Pickering & Garrod, 2013). As argued in Pardo et al. (2018), this leads to the prediction that any task that measures shifts in speech production after listening to a talker with different acoustic characteristics should show a similar pattern, since listening to that talker should result in similar covert articulation of perceived speech, whatever the task at hand. Extending this argument to deliberate vs. spontaneous imitation behaviors, any listener who shows a high degree of spontaneous convergence should show a similarly high degree of deliberate imitation ability, although this account leaves open the possibility that factors beyond this perceptual-motor loop might influence overt imitation behavior. Indeed, spontaneous and deliberate imitation must share some mechanisms, since the same perceptual system is involved in perceiving differences in the speech of talkers, and deliberate and spontaneous imitation behaviors are both implemented by the same speech motor system. What is unclear, however, is the extent to which *individual differences* in these behaviors are predicted by the same factors. Schertz et al. (2023) characterize deliberate speech imitation as the summation of an automatic imitation mechanism combined with perceptual and articulatory skills that control the performance of imitation which vary by individual. We might add to this characterization that deliberate imitation is additionally likely to be modulated by social factors such as one’s attitude towards the imitated talker (Adank, Rueschemeyer, & Bekkering, 2013). Equally, however, one might characterize *spontaneous* imitation behavior as resulting both from individual differences in underlying capacity to imitate (limited by perceptual, motor, cognitive, and personality factors) as well as one’s propensity to engage in automatic imitation in a given social context.

Curiously, few studies exist to allow us to directly test the hypothesis that individual differences in spontaneous and deliberate imitation lean on the same underlying set of skills within the same set of participants. In a study by Sato et al. (2013), participants performed a spontaneous phonetic convergence task on pitch-shifted French vowels, followed by instructions to explicitly imitate these materials. The degree of production change over the two tasks did not correlate strongly, but because the sample represented a subset (*n* = 12) of the total sample, it may be that this smaller sample size was not sufficient to allow a weak relationship to emerge. An fMRI study from the same group (Garnier et al., 2013) measured brain activity in native French speakers during both spontaneous and deliberate imitation of pitch-shifted French vowels. Due to correlations between activation in the dorsal stream and imitation ability, they proposed that these two skills rely on the same underlying network, although it would also be possible to attribute these patterns to overlap in other aspects of the task (e.g., shared motor output skills). Though the authors report individual-level behavioral data that is suggestive that better deliberate imitators are also more apt to automatically imitate speech, they do not explicitly analyze this relationship.

Other studies use a between-subjects approach to investigate the degree of acoustic shift either when participants are explicitly asked to imitate speech or when they are in a spontaneous imitation paradigm. In general, these studies show that different groups of participants, when exposed to the same phonetic materials, will show qualitatively similar (although quantitatively distinct) shifts in production whether or not they are given instructions to imitate (Clopper & Dossey, 2020; Delaney et al., 2010; Dufour & Nguyen, 2013). However, these studies cannot speak to whether individual differences in imitation are consistent across deliberate vs. spontaneous tasks since they use between-subjects samples. Although not a study of deliberate imitation, Pardo et al. (2018) report a within-subject design with a robust sample size (90 participants completed both tasks) where participants engaged in a spontaneous conversational convergence task in one session, then returned for a shadowing task. Here the relationship between the degree of shift between conversational and shadowing tasks was somewhat inconsistent, and modulated by talker sex. One likely reason for the lack of studies using both spontaneous and deliberate tasks is that once one gives a participant instructions to imitate, they can’t “unknow” those instructions when they are then placed in a spontaneous imitation task, which creates problems for counterbalancing tasks.

An alternative approach to investigating the similarity between individual differences in spontaneous vs. deliberate imitation is to probe the degree to which skills, traits, and capacities that have been found to predict individual differences in spontaneous imitation also predict deliberate speech imitation. As we discuss below, a number of studies have investigated predictors of vocal imitation in both spontaneous and deliberate imitation tasks. Studies of speech alignment tend to use experimental paradigms in which participants are not explicitly instructed to align speaking styles and then factors such as social cohesiveness, shared goal, or materials are manipulated to observe whether talkers tend to align (see above).

In this exploration, we include multiple potential predictors of deliberate imitation skill to investigate both known factors underlying imitation and new measures of individual differences that may lead to new insights. We extend theories of spontaneous imitation to predict that social context (affinity to the talker), and personality type, as well as perceptual and articulatory skill will be associated with deliberate imitation. To be clear, finding that the same predictors of spontaneous imitation also predict deliberate imitation does not allow us to conclude that these are one and the same mechanism, but rather highlights potential overlap and similarity among these processes. Further, although a few predictors have been investigated across spontaneous and deliberate imitation paradigms—for instance, “affinity” to the model talker has been shown to modulate both spontaneous and deliberate imitation (Adank, Stewart, et al., 2013; e.g., Babel, 2010)—studies that look for relationships between a comprehensive set of perceptual, motor, cognitive, and personality predictors in one well-powered sample are rare (e.g., Reiterer et al., 2013; Yu et al., 2013). The value to collecting many predictors within the same study exceeds “more is better”—by examining many predictors of imitation ability in the same sample, we can identify patterns of co-occurrence among measures and investigate which uniquely predict imitation ability after accounting for the other factors. There is increasing interest in exploring how individual differences in language production and perception pattern in the population and how these are predicted by language-internal differences (e.g., structure of the phonetic category) as well as language-external factors (e.g., working memory; Kim & Clayards, 2019; Heffner & Myers, 2021; see Yu & Zellou, 2019 for review). Discovering the co-occurrence (and potential co-dependence) of sets of predictors advances the goal of characterizing the sources of individual differences in language.

Below we review studies that have looked for predictors of individual differences in vocal imitation, whether in spontaneous or deliberate paradigms. Each category of predictors is broadly motivated by a different theory of vocal imitation, divided below into perceptual-motor factors, shared linguistic representations, social and personality predictors, and cognitive predictors. Notably, these theories are not designed to account for individual differences in imitation, per se, however, to the extent that each implicates a set of component processes (e.g., speech representations, perceptual-motor skills, social sensitivity), variability in these factors should also predict imitation ability.

### Perceptual-Motor Predictors of Imitation

Some theories account for phonetic alignment by appealing to perceptual-motor mechanisms, where, as communication partners take turns producing and perceiving speech, their individual motor and perceptual representations begin to align (Moulin-Frier et al., 2015; Nguyen & Delvaux, 2015). These accounts have their roots in animal models of perception and action (Mercado et al., 2014), often with reference to so-called “mirror” systems that are proposed to facilitate automatic transfer of observed behavior into imitated behavior. This view is consistent with data showing that practice with deliberate imitation of accented speech leads to more accurate perception of that accent, perhaps due to a better-elaborated perceptual-motor representation of the characteristics of the accent after practice with imitation (Adank et al., 2010; Evans & Iverson, 2007). Similarly, musicians, who are likely to have enhanced auditory perceptual skills (Skoe & Kraus, 2013), outperform non-musicians on deliberate imitation of an unknown language (Turker et al., 2017), and those with vocal training outperform instrumentalists (Christiner & Reiterer, 2013, 2015), suggesting that variability in perceptual and motor expertise (that is enhanced in musicians) may be a limiting factor on vocal imitation ability.

### Shared Linguistic Representations

A cousin to the perceptual-motor account suggests that speech alignment results from automatic priming of shared speech representations at multiple levels of representation without necessarily being driven by social factors (Pickering & Garrod, 2004; although see Gambi & Pickering, 2013 for an incorporation of social context into this framework). This view would predict that perceptual sensitivities to a specific speech contrast should mirror production ability. In a study of explicit imitation of voice-onset time (VOT) contrasts, Kim and Clayards (2019) show that the cue weights that listeners assign in a perceptual task relate to their use of those same cues in production, suggesting access to a common perceptual and motor representation during imitation. Schertz et al. (2023) report that discrimination of target accents is related to explicit imitation of those same accents, suggesting that individual differences in processing the acoustic-phonetic qualities of accented speech support production. An emerging literature suggests that, aside from alignment in perception and production of specific phonetic contrasts, listeners differ substantially in their perceptual sensitivity to phonetic contrasts (Fuhrmeister et al., 2023; Kapnoula et al., 2017), with some showing more fine-grained sensitivity to contrasts within the phonetic category and others showing more classically “categorical” patterns where within-category contrasts are difficult to distinguish. In theory, listeners with more sensitive acoustic-phonetic spaces would be better positioned to detect these differences in a speech sample in order to reproduce them in production, a question that has yet to be tested.

### Social and Personality Predictors of Imitation

Communication Accommodation Theory centers the *social* function of vocal imitation, suggesting that social alignment is the primary goal of speech convergence (Giles et al., 1991). The notion that social factors (i.e., one’s disposition towards the talker) modulate imitation finds ample support within the spontaneous imitation literature. Spontaneous alignment with conversation partners or to speech we are exposed to increases when we have an affinity to our interlocutor (Babel, 2010; Babel et al., 2014; Pardo et al., 2012). For instance, Pardo and colleagues showed that the speech patterns of college roommates became more similar to one another over time, an effect that was related to the degree of closeness reported by the pair (Pardo et al., 2012). The degree of convergence may also depend on attitudes towards the social or cultural group a talker belongs to. Babel (2010) reported that speakers of New Zealand English showed more spontaneous convergence with an Australian-accented talker when they reported a pro-Australian bias and, in a separate study, the degree of convergence with a pictured talker was related to judgments of that talker’s attractiveness (Babel, 2012), and Yu and colleagues report greater spontaneous phonetic alignment when the talker reports a more positive attitude towards the model talker (Yu et al., 2013). Social alignment effects of vocal imitation may even be bi-directional. Adank and colleagues showed that participants who were asked to imitate an accent reported more favorable ratings of the talker’s “social attractiveness” (Adank, Stewart, et al., 2013).

Affinity for the specific model talker is one factor modulating imitation, but one’s general social disposition or personality may also play a role. Aguilar et al. (2016) showed that participants who scored highly on a scale of trait rejection sensitivity showed more convergence in a spontaneous imitation task, suggesting that a disposition towards social acceptance-seeking may enhance alignment. Conversely, those who score more highly on an autism questionnaire show less alignment in a spontaneous imitation task (Snyder et al., 2019), pointing to individual differences in social behavior as a modulator of imitation. Personality factors have also been shown to affect spontaneous imitation–work by Yu et al. (2013) reported that talkers who scored highly on trait “Openness” as measured by the Big Five personality inventory (John et al., 1991), showed greater lengthening of VOTs for voiceless stops after having listened to a narrative from a talker with lengthened VOTs (see also Schertz et al., 2023). They posited that Openness, in this case, represented a willingness to engage with the new phonetic information. Similarly, Lewandowski and Jilka (2019) asked native German speakers who had learned English as a second language to work together with native American and British English speakers to complete “spot-the-difference” games and examined their conversations, finding that participants with higher Openness scores exhibited a higher degree of spontaneous phonetic convergence with the accented talkers. Notably, these studies all used an implicit measure of imitation—participants were not instructed to imitate the voice they had heard. In this sense, it is unclear whether trait openness is more related to one’s propensity to align with another talker, or to one’s ability to imitate.

### Cognitive and Linguistic Factors

Individual differences in cognitive abilities and general (i.e., non-phonetic) linguistic ability are not firmly linked to a specific theoretical stance on vocal imitation. Nonetheless, domain-general cognitive factors such as attention and working memory, and factors that reflect broad linguistic experience such as vocabulary size may theoretically modulate the more domain-specific perception-production process involved in imitation. Janse and Adank (2012) identified vocabulary size and selective attention as predictors of perceptual adaptation to accented speech over time, speculating that individuals with rich vocabularies may be better able to pinpoint systematic differences between a non-native accent and their own. Yu et al. (2013) similarly showed that attention switching was related to spontaneous imitation. The ability to imitate speech may also depend on the ability to hold speech in memory; Reiterer et al. (2011) showed a correlation between deliberate imitation of an unknown language and digit span, a finding replicated in Christiner and Reiterer (2015). Thus, it seems likely that domain-general cognitive traits modulate imitation ability; nonetheless, the degree to which these traits co-vary with other skills and traits also thought to predict imitation is unknown.^1^

### The Current Study

**Figure 1.**
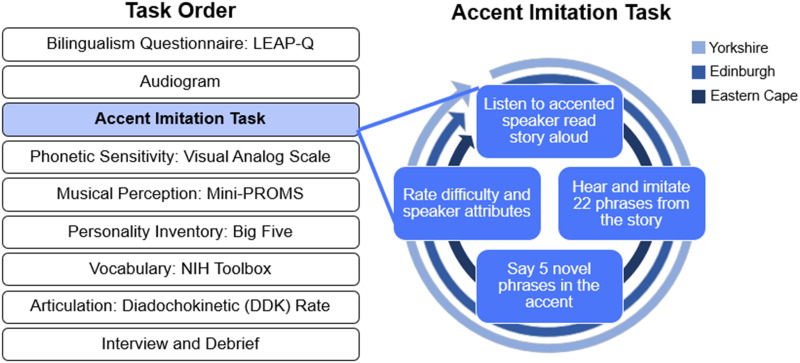
Experiment schematic. Tasks were administered in order from top to bottom. See text for a description of each task or measure.

In this study, we asked which individual characteristics best predict accent imitation ability. Inspired by theories of speech alignment, we predicted that the perceptual and motor skills of listeners would correlate with accent imitation ability. However, recognizing the social role that voice mode plays, we also predicted that personality, particularly openness (Schertz et al., 2023; Yu et al., 2013), and affinity to the model talker (Adank, Stewart, et al., 2013; e.g., Babel, 2010) would modulate this behavior. Although several studies have investigated musicianship and musical skill as a predictor of deliberate imitation (Christiner & Reiterer, 2013, 2015; Turker et al., 2017) and at least one study has looked at talker attitudes as a driver of deliberate imitation ability (Adank, Stewart, et al., 2013), to our knowledge, no study has tested articulatory speed, personality, and phonetic perception ability as drivers of individual differences in deliberate imitation. To this end, we recruited a large sample (*n* = 92) of participants from the University of Connecticut and community and recorded their attempts to imitate short phrases taken from three accents. We assessed a range of perceptual, motor, cognitive, and personality dimensions (see Figure 1). We tested fine-grained sensitivity to phonetic detail using a speech sound rating task, and musical perception was evaluated using an objective measure that taps melody, tuning, rhythm, and timing processing (Zentner & Strauss, 2017). Articulatory speed was measured using a speech production task that resembles a tongue-twister. Vocabulary size was assessed using an adaptive picture vocabulary test (Gershon et al., 2014), and personality variation was measured using the Big Five Inventory, which produces scores on dimensions of Extraversion, Neuroticism, Conscientiousness, Openness, and Agreeableness (John et al., 1991). Accent imitation attempts were perceptually rated by an independent set of raters from an online participant pool (Prolific) and we used multiple linear regression to find the set of individual characteristics that best predict accent imitation ability. To the extent that any measure predicts individual differences in explicit imitation, it should be considered as a candidate variable in theoretical models of deliberate vocal imitation.

## METHODS

### Participants

#### Imitators.

Adult native speakers of English (*n* = 92) were recruited from the University of Connecticut community and environs. Participants used North American dialects of English to interact with the study team. The age range was 18–58 (mean = 20.86, *SD* = 6.84), and the sample included 60 women and 32 men. In addition to a broad community sample, recruitment efforts targeted drama students and professional actors who have had training in accent imitation, including 6 professional actors and 10 drama students. 45 participants were categorized as musicians, as defined by the relatively lenient criteria that they sang or played an instrument. 22 participants were monolingual, reporting that they spoke only English. All participants gave informed consent according to the guidelines of the University of Connecticut Institutional Review Board, and were compensated either with course credit or at a rate of $11 per hour.

### Procedures

After consent, participants completed a series of tasks within a 90-minute testing session, in the following order: a modified version of the Language Experience and Proficiency Questionnaire (LEAP-Q) to assess bilingualism (Marian et al., 2007), a pure-tone audiogram, the accent imitation task, a visual analogue scale test of speech perception, the mini version of the Profile of Music Perception Skills (mini-PROMS) to assess musical perception (Zentner & Strauss, 2017), the Big Five Inventory (John et al., 1991), the NIH Toolbox picture vocabulary test (Gershon et al., 2014), a diadochokinetic (DDK) test of articulatory rate, a short interview about experience with vocal performance and code-switching, and a debriefing. Each task is described in more detail below.

#### Accent Imitation Task.

Non-US accents of English were selected for imitation purposes. We chose accents that were less likely to be familiar to our participant population, and that are not generally associated with negative stereotyping in US society. All model speakers used native dialects of English (rather than non-native dialects). Audio samples were used with permission from the International Dialects of English Archive (IDEA), and materials consisted of speakers reading the “Comma Gets a Cure” story (Honorof et al., 2000), a short story that is constructed to sample from a wide variety of vowels and consonants of English. Model speakers were one male speaker from Yorkshire, England, one female speaker from Edinburgh, Scotland, and one female speaker from the Eastern Cape region of South Africa. The precise acoustic differences between these accents, and the acoustic characteristics of the imitated samples, are of substantial interest but beyond the scope of the current report.

During the accent imitation task, participants were asked to imitate the accents of three different talkers. The task consisted of three blocks, one for each accented talker, and the order of the accents was counterbalanced across participants. First, participants passively listened to the entire “Comma Gets a Cure” story to familiarize themselves with the voice. Then they heard 18 shorter phrases (5–8 words) and 4 longer phrases (9–14 words) excerpted from the story. Participants heard one phrase at a time and heard the sample phrase from the story once before recording their attempt to imitate the phrase. At the end of these 22 imitation phrases, participants moved to a “generalization” phase, in which they saw a novel written phrase (not included in the audio sample) and were asked to say it in the accent they had just imitated. Generalization phrases were not included in the subsequent analysis since they could not be submitted to the same rating procedure, as there was no “correct” model for these phrases. These phrases were checked for audio completeness (i.e., not cut off during recording) and, in the event that the participant recorded more than one attempt at the phrase, we edited the recording to include only the second attempt. Due to lab shutdowns in 2020, only the first 17 phrases were able to be processed to submit to raters. Every participant had at least 11 of 17 intact recordings for each accent for rating purposes (*M* = 16.65, *SD* = 0.98).

Following each block, participants were asked a series of questions about the accent they had just heard. First, they rated how difficult the accent was to imitate on a five-point scale. Next, following methods from prior studies (Adank, Stewart, et al., 2013; Bayard et al., 2001), they rated the talker’s voice according to a number of attributes, including friendliness, attractiveness, authoritativeness, etc. (See Appendix A for this questionnaire).

#### Accent Ratings.

There are many possible methods for assessing each participant’s success in accent imitation, including expert judgments or acoustic measurements (see Pardo, 2013 for a discussion of the many possible acoustic measurements and their convergence with perceptual ratings). In order to capture as many possible features of the imitated accent as possible, an independent set of raters judged the degree to which each accented sample approximated the intended target. We recruited raters from the online participant resource, Prolific. The study was advertised to adult participants who were native speakers of English living in the US. Participants gave informed consent and were compensated at $11 per hour. Before proceeding to the accent rating task, participants first did a headphone check (Woods et al., 2017) to ensure that they were using headphones.

During the accent rating task, participants were instructed to rate the similarity of imitated phrases to the accented phrase in terms of the accent specifically (and not other qualities of the voice) on a seven-point scale, with one being least like the accent and seven being most similar to the accent. Each rater judged 50 distinct talkers’ attempts to imitate only one accented phrase (e.g., “near the Duke Street tower” in the Yorkshire accent, as produced by 50 imitators), which enabled listeners to develop some familiarity with that specific phrase, and to get a sense of the range of abilities of the imitators. To check for raters who might not be performing the task in good faith (e.g., were pressing one response button indiscriminately), we searched for any participant who used the same rating value more than 35 times (out of 50). Luckily, no raters met this criterion, so all raters were included. A few raters (*n* = 5) accidentally participated twice. One session for each repeated subject was dropped at random. In total, *n* = 1026 participants contributed ratings. Finally, in order to make the ratings lists of equal size, some imitation attempts received more ratings than others. We capped the number of ratings per attempt at 11 by random selection of ratings. As such, each imitation attempt received at least *n* = 6 and as many as *n* = 11 ratings (mean = 10.05, *SD* = 0.50) from online participants.

#### Individual Differences Battery.

Individual differences measures included a set of tests assessing language history, hearing status, personality, vocabulary, and musical perception skill. We list each below.

- **Bilingualism questionnaire**. Participants completed a shortened version of the LEAP-Q measure of bilingualism, in which they were asked to report the languages they spoke in order of acquisition as well as how often they chose to use each language in certain situations, such as reading and spoken conversations with others (Marian et al., 2007).
- **Pure-tone audiometry**. All participants had an average pure-tone threshold of 30 dB or better in both ears, testing at 0.5, 1, 2, 3, 4, 6, and 8 kHz.
- **Personality inventory**. The Big Five personality inventory was administered (John et al., 1991). This inventory provides self-report scores on measures of five key personality variables: Agreeableness, Conscientiousness, Extraversion, Neuroticism, and Openness. Participants used a 5-point Likert scale to rank how much they agreed or disagreed that statements related to the five personality factors generally described them.
- **Vocabulary**. Vocabulary was measured using an adaptive picture vocabulary test (NIH Toolbox) administered on an iPad mini (Gershon et al., 2014). Participants were instructed to select which one of four images on the screen best matched the definition of each word. They heard a recording of a vocabulary word spoken aloud and then tapped the screen to give their response.
- **Musical perceptual skill**. The Mini-PROMS test is an objective measure of perceptual skills for music and includes subtests that measure tuning (being able to tell whether a note is in-tune or out of tune in a chord), melody (discrimination of similar melodic passages), beat (discriminating rhythmic patterns), and tempo [judging differences in overall rate of musical passages; Strauss et al., 2015; Zentner & Strauss, 2017]. Participants filled out a questionnaire about their musical training experience and then completed discrimination tasks for each subtest in which they heard two short musical recordings and reported their decision and confidence level on a 5-point scale (definitely different, probably different, I don’t know, probably same, definitely same).
- **Articulatory ability**. Diadochokinetic rate, or DDK, tests an individual’s maximum speech rate. This test is sometimes used clinically to assess speech production disorders (Kent et al., 2022; Maas, 2017), and also correlates with executive control abilities in adults (Shen & Janse, 2020). Participants were asked to repeat a syllable or set of syllables (/pa/, /ta/, /ka/, and /pataka/) as quickly as possible for five seconds. These efforts were audio recorded and scored off-line for the number of repetitions in five seconds. Higher scores indicate higher maximum speech rate.
- **Phonetic sensitivity**. As an exploratory measure, we tested whether individual differences in native language speech sound perception predicted accent imitation, with the rationale that the ability to detect fine-grained phonetic differences might support perceptual learning of the accent. Using a variant of the “visual analogue scale” rating task (Fuhrmeister & Myers, 2021; Kapnoula et al., 2017), participants heard items randomly selected from along a synthetic continuum from /ba/ to /da/ and for each token indicated how “ba-like” or “da-like” each token sounded along a 7-point scale. To estimate the sharpness of the psychometric response function, we ran a mixed effects non-linear regression using the *nlme* package (Pinheiro & Bates, 2000) which fit the ratings to a two-parameter logistic function, estimating coefficients for the inflection point (the category boundary), and the slope of the function. Larger values indicate steeper slopes and a more categorical-like response function, whereas smaller values indicate more graded perceptual sensitivity. We also estimated “response consistency,” the degree to which participants consistently assigned the same rating value to the same point along the phonetic continuum, by computing the average of the standard deviations of the ratings for each continuum point for each subject.
- **Interview**. At the end of the experimental session, we conducted a short open-response interview to collect qualitative data about other types of experiences with voice use or voice switching, including vocal performance (singing and acting) affinity and experience, experiences with social or cultural code-switching, and voice experiences related to gender and sexual identity. These interviews are of interest but beyond the scope of the current report.

### Data and Code Sharing

Data and code for this project are available at on the Open Science Framework: https://osf.io/8erq3/.

## RESULTS

### Accent Ratings: Descriptive Statistics

**Figure 2.**
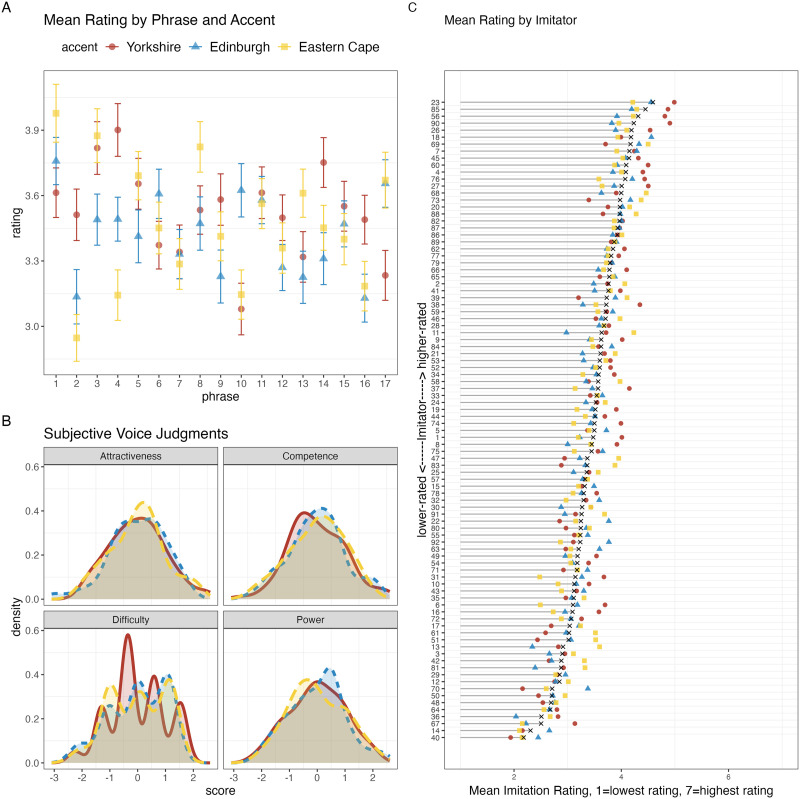
A. Mean accent imitation ratings by phrase. B. Subjective judgements on the part of the imitators regarding the perceived attractiveness, competence, and powerfulness of the imitated talker, as well as self-rated difficulty of the imitation attempt. C. Imitators ranked according to the mean rating of imitation accuracy on the part of independent raters.

To check if certain phrases or accents were particularly difficult or easy to imitate, we calculated the mean attempt rating for each subject by phrase and accent. Results are displayed in Figure 2. Visual inspection suggests a substantial range in accent imitation ratings, with the best-rated imitator achieving a score of 4.99 and the lowest-rated imitator a score of 1.94 (on a scale from 1–7, Figure 2C). Similarly, the imitation ratings differed by phrase and accent (Figure 2A).

Prior work has suggested that the quality of vocal mimicry might depend partially on the degree to which participants entertain positive feelings about members of the accent group in general, or about the talker in particular (e.g., Babel, 2010). Following methods from Bayard et al. (2001, see also Adank, Stewart, et al., 2013), we calculated the mean response on questions measuring rankings of the “power,” “competence,” and “social attractiveness” for each accent, and also included subjects’ subjective rating of how difficult each accent was to imitate (Figure 2B, see Appendix A for a list of the question prompts).

### Predicting Accent Imitation Scores From Individual Differences Measures

**Figure 3.**
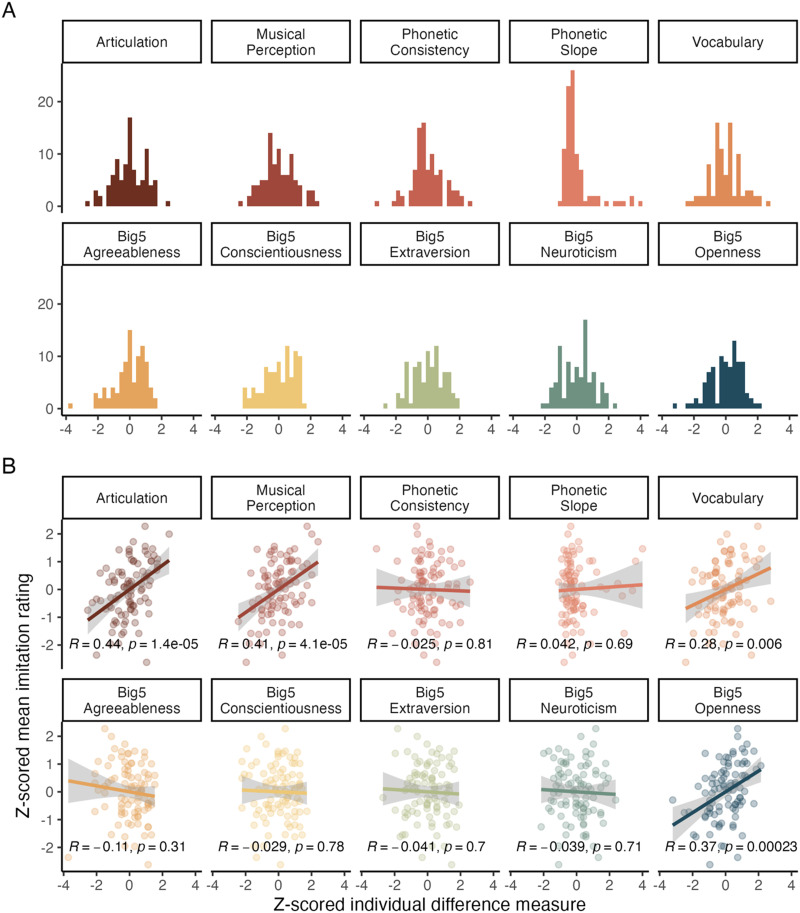
Individual differences measures and their relationship to the imitation rating score. A. histogram of individual differences measures, z-scored. B. Relationship between the z-scored measures and the mean by-participant imitation scores, across all accents. Pearson correlation coefficients (uncorrected for multiple comparisons) displayed for descriptive purposes.

All cognitive, perceptual, and personality measures were z-scored. Histograms displaying the distribution of these measures are displayed in Figure 3A. For descriptive purposes, correlations between all individual difference scores were calculated using Pearson correlations (Table 1), and uncorrected correlations between individual differences measures and imitation ratings are displayed in Figure 3B.

**Table 1.** Correlations between individual differences measures. Pearson correlations displayed, with significance values (uncorrected) listed for descriptive purposes.

	1	2	3	4	5	6	7	8	9	10
1. Imitation Score	–									
2. Big5 Agreeableness	−.11	–								
3. Big5 Conscientiousness	−.03	.29**	–							
4. Big5 Extraversion	−.04	.00	.17	–						
5. Big5 Neuroticism	−.04	−.17	−.26*	−.28**	–					
6. Big5 Openness	.37***	.02	−.09	.13	−.12	–				
7. Musical Perception	.41***	−.09	.02	−.08	.01	.36***	–			
8. Articulation	.44***	−.16	−.07	.06	−.15	.30**	.33**	–		
9. Phonetic Consistency	−.03	−.01	−.04	.04	.05	−.06	−.01	−.24*	–	
10. Phonetic Slope	.04	.08	.12	.12	.05	.14	.03	.17	−.53***	–
11. Vocabulary	.28**	−.32**	−.05	.02	−.04	.34***	.30**	.23*	−.19	.03

*Note*. *p < 0.05; **p < 0.01; ***p < 0.001.

#### Regression and Model Selection.

We built a linear mixed-effects model using the *lme* function from the *lme4* package in R (Bates et al., 2015). The full model predicted each individual attempt rating using as predictors scaled measures of Musical Perception (Mini-PROMS), Agreeableness, Conscientiousness, Extraversion, Neuroticism, Openness (Big Five Inventory), Vocabulary (NIH Toolbox), Articulation (mean DDK rate), Phonetic Categorization Consistency, Phonetic Categorization Slope, as well as accent-specific imitator judgments of Difficulty of imitation, and subjective judgments of the Competence, Attractiveness, and Powerfulness of each imitated voice. Further, we included subject demographics, including Age, self-reported Sex, Bilingualism, and Musical training. We also included random intercepts by Participant (the imitator), Accent, Rater, and Phrase. (Full model syntax: rating ∼ Age + Difficulty + Power + Competence + Attractiveness + Sex + Bilingual + Musician + BigFiveAgreeableness + BigFiveConscientiousness + BigFiveExtraversion + BigFiveNeuroticism + BigFiveOpenness + Vocabulary + MusicalPerception + Articulation + PhoneticConsistency + PhoneticSlope + (1 | Participant) + (1 | Accent) + (1 | Rater) + (1 | Phrase); output tables from the full model are displayed in Appendix B). Model selection was performed using a backwards-stepping procedure implemented using the *step* function from the *lmerTest* package (Kuznetsova et al., 2017). This procedure iteratively removes low-performing predictors from the model, first removing random-effect terms, then fixed-effect terms and compares the simpler model to the more complex model until a best-fit model is found. The default method of computing denominator degrees of freedom (Satterthwaite) was employed.

The best-fit model included participant judgments of the difficulty of imitating the accent (Difficulty), the items from the subjective ratings data reflecting imitator ratings of the powerfulness and competence of the voice (Power, Competence), the personality trait of Openness (BigFiveOpenness), the results of the mini-PROMS (MusicalPerception) and the articulatory rate as measured by DDK (Articulation). Further, random effects of the participant, the rater, and the phrase contributed to model fit (Model syntax: rating ∼ Difficulty + Power + Competence + BigFiveOpenness + MusicalPerception + Articulation + (1 | Participant) + (1 | Rater) + (1 | Phrase)). Results of this best-fit model are displayed in Table 2.

**Table 2.** Best-fit model predicting accent imitation accuracy from a set of continuous and categorical predictors.

Term	$\hat{\beta }$	95% CI	*t*	*df*	*p*
Intercept	3.48	[3.36, 3.59]	60.37	63.11	<.001
Difficulty	−0.06	[−0.08, −0.04]	−5.75	36,424.35	<.001
Power	−0.04	[−0.06, −0.02]	−3.69	39,955.20	<.001
Competence	0.05	[0.03, 0.08]	4.53	32,248.29	<.001
BigFiveOpenness	0.12	[0.02, 0.21]	2.40	86.70	.018
MusicalPerception	0.13	[0.04, 0.23]	2.70	86.94	.008
Articulation	0.13	[0.04, 0.22]	2.73	86.83	.008

First, considering the relationship between subjective ratings and imitation success, there was a negative relationship between participant judgments of the difficulty of the voice to imitate, and ratings of imitation success, such that when a participant found the voice more difficult to imitate, their attempts were rated lower by independent raters. The imitator’s judgment of two aspects of the individual voices also predicted ratings of their imitation attempts. Judgments of the degree to which the voice was considered “powerful” were negatively correlated with imitation ratings, whereas the degree to which the voice was considered “competent” was positively related. No other subjective voice judgments predicted later imitation ratings (refer to Table 2).

Turning to the individual differences measures and their relationship to imitation ratings, three measures survived model selection. Specifically, there was a positive relationship between better scores on the Mini-PROMS test of musical perception and better imitation scores. Our articulation measure (DDK) was also a strong predictor, with individuals who produced more syllables per second also receiving higher ratings on their imitation attempts. The relationship with Openness was likewise positive. Relationships between all predictors and the accent imitation score are displayed in Figure 3B.

## DISCUSSION

People differ substantially in their ability to imitate other voices. Notably, individual differences in accent imitation ability were significantly related to several component skills or traits. After model selection, differences in accent imitation ability were predicted by musical perception ability (as measured by the Mini-PROMS), articulatory agility (as measured by a diadochokinetic task), and the personality feature of “openness” (as measured by the Big Five Inventory), as well as by two subjective ratings of the to-be-imitated talker on the part of the imitator, the degree to which they are judged as “powerful” as well as “competent.”

Before discussing each of these predictors individually, it is important to note that these skills, traits, and experiences do not pattern randomly in our dataset. Indeed, there was significant covariance shared between several predictors, especially musical perception ability, articulation, vocabulary, and openness (see Table 1). Any study measuring individual differences in the wild must grapple with the co-occurrence of distinct traits and skills within individuals. In the current sample, for instance, we have no way of knowing whether openness in personality taps a stable trait that prepares the listener to be sensitive to acoustic detail and ready to imitate those qualities, or if (for instance) openness in personality is simply correlated with opportunities to experience diverse accents. From a statistical perspective, the collinearity of many traits also can obscure the contribution of any one measure to the predictor variable. Nonetheless, factors we identified that predict accent imitation skill can be easily interpreted in light of the underlying processes that are necessary for accent imitation.

Accent imitation is an interesting domain to study precisely because the task cuts across a large number of perceptual and motor skills (auditory acuity, speech perception, motor agility) cognitive components (working memory, language fluency), and even personality traits. By measuring individual differences in these many components, we have the opportunity to sketch a more complete picture of the interlocking pieces of accent imitation. Importantly, as we discuss below, our results show that successful accent imitation rests on a foundation of apparently prerequisite perceptual and motor skills (musical perception, articulatory ability) but is also significantly related to trait openness, as well as the imitator’s disposition towards the imitated talker.

### Perceptual-Motor Skills Related to Accent Imitation

Musical perception skills were a strong predictor of accent imitation ability. Musicians may have more finely honed abilities in auditory processing (either due to training, or even emerging from inherent perceptual skill) that allow them to more accurately perceive the fine-grained acoustic details that mark the accent and attend to subtle differences in speech (e.g., Skoe & Kraus, 2013). The relationship between musical skill and deliberate imitation of non-native speech has been demonstrated in adults (Christiner & Reiterer, 2015; Turker et al., 2017) and even in children as young as five years old (Christiner & Reiterer, 2018). Subtests of the Mini-PROMS tap participants’ ability to discriminate small differences in both melody and rhythm, skills that may be transferrable to accent imitation. Of interest, although musical perception related to accent imitation, a more specific measure of phonetic perception, namely consistency and slope in the VAS task, did not predict imitation ratings. We speculate that fine-grained sensitivity to segmental differences (at least for this particular contrast: b/d) may be less useful than more general abilities in tracking rhythm and pitch.

In accent imitation, the listener must also become a speech producer—that is, must translate the regularities extracted from the input into movements of the speech articulators. Our test of speech articulation agility involved rapid production of consonant-vowel syllables (e.g., *pataka*)–a task that is fairly distant from the accent imitation task, and which emphasized speed rather than perfect accuracy. In our study, articulation ability (as estimated from the mean DDK rate) was a significant predictor of accent imitation, suggesting that the ability to implement those perceptual models in speech articulation is key. This finding is consistent with prior work showing that among musicians, people with vocal training outperform instrumentalists (Christiner & Reiterer, 2015) in deliberately imitating an unfamiliar language, suggesting that strong vocal motor skills provide a foundation for imitation ability. The finding that individual differences in perceptual and motor skill are substrates for better imitation ability is consistent with models of spontaneous phonetic alignment that focus on the perceptual-motor circuits that coordinate auditory input with motor output and self-monitoring (Moulin-Frier et al., 2015). If the results of the current study represent a limit on the ability of a participant to imitate speech at all (whether automatically-triggered or deliberately initiated), then individuals with stronger domain-general auditory perception skills (musical perception ability) and stronger speech-motor skills (DDK rate) would be predicted to show greater phonetic alignment in spontaneous imitation tasks, a prediction that has, to our knowledge, yet to be tested.

### Social and Personality Variables Predicting Imitation Ability

Given the role of speech generally, and mimicry specifically, in complex social dynamics (Giles et al., 1991), we considered whether differences in personality type—differences that guide how people relate to one another—might also predict imitation behavior. One component of the Big Five Inventory, Openness, was predictive of imitation skill. People who endorse questions like “is curious about many different things,” “has an active imagination,” and “likes to reflect, play with ideas” score highly on this scale, which is thought to reflect receptiveness to new experiences. Of interest, people in the arts, including actors, often score highly on this scale (Silvia, 2007). Notably a number of items on this scale also specifically refer to artistic pursuits, including “values artistic, aesthetic experiences” and “is sophisticated in art, music, literature,” as well as a reverse-scored item “has few artistic interests.” This calls into question whether the relationship with accent imitation is by virtue of variation in one’s willingness to try new things—in this case, try on a new accent—or rather just a participant’s engagement with the arts, which might bring them into contact with a wider variety of types of voices. This finding aligns with others showing that personality plays a mediating role in predicting the amount of spontaneous alignment that an individual exhibits, with participants with greater openness (Lewandowski & Jilka, 2019; Yu et al., 2013), more rejection sensitivity (Aguilar et al., 2016) and higher neuroticism (Lewandowski & Jilka, 2019) showing greater alignment. Notably, neuroticism did not predict imitation ability in our findings. We speculate that trait neuroticism may be more relevant for imitation when a conversation partner is present (as in Lewandowski & Jilka, 2019), and may reflect individual differences in a participant’s sense of power balance between communication dyads. The fact that personality also predicts imitation under directed conditions suggests that these personality factors are not only a prediction of the disposition of the imitator to the target talker, but that personality differences may drive differences in imitation skills in a more general way.

As in numerous studies of spontaneous imitation, (Babel, 2012; Stel & Vonk, 2010) positive disposition towards the imitator predicted imitation success. Participants who judged the to-be-imitated voice as more “competent” showed greater success in imitation, whereas rating a particular talker as more “powerful” was negatively related to imitation success (an unanticipated finding). With respect to this latter finding, previous work has shown that in dyadic pairs, an asymmetry in perceived power may lead to more convergence on the part of the less powerful conversation partner (e.g., Gregory & Webster, 1996). In the current context, since there is only one “live” communication partner, it may be that measures of “power” are less relevant, since there is no role of deference or dominance in the interaction. It may be, instead, that our respondents (primarily college students) interpret the “powerful” item as a measure of social distance, and instead are less inclined to imitate a voice that is dissimilar from their own. In a general sense, judgments about the imitated voice may be made on different criteria in this study than in those where a conversation partner is present in the room. Similarly, we failed to reproduce prior studies showing that judgments of talker “attractiveness” predict spontaneous imitation (Babel, 2012). However, it may be consequential that we asked for these ratings after the imitation attempt. Given reports that engaging in deliberate accent imitation itself increases one’s positivity towards the talker (Adank, Stewart, et al., 2013), any relationship between affinity towards the talker and imitation skill might be more difficult to measure after the talker has engaged in imitation. This study was not designed to test how factors such as perceived talker gender, sociolinguistic factors about the perceived status of the dialect in general (e.g., Bayard et al., 2001), or other inferred details of the talker might affect imitation success, which remain an interesting question for future study.

### A Synthetic Model of Imitation Ability

**Figure 4.**
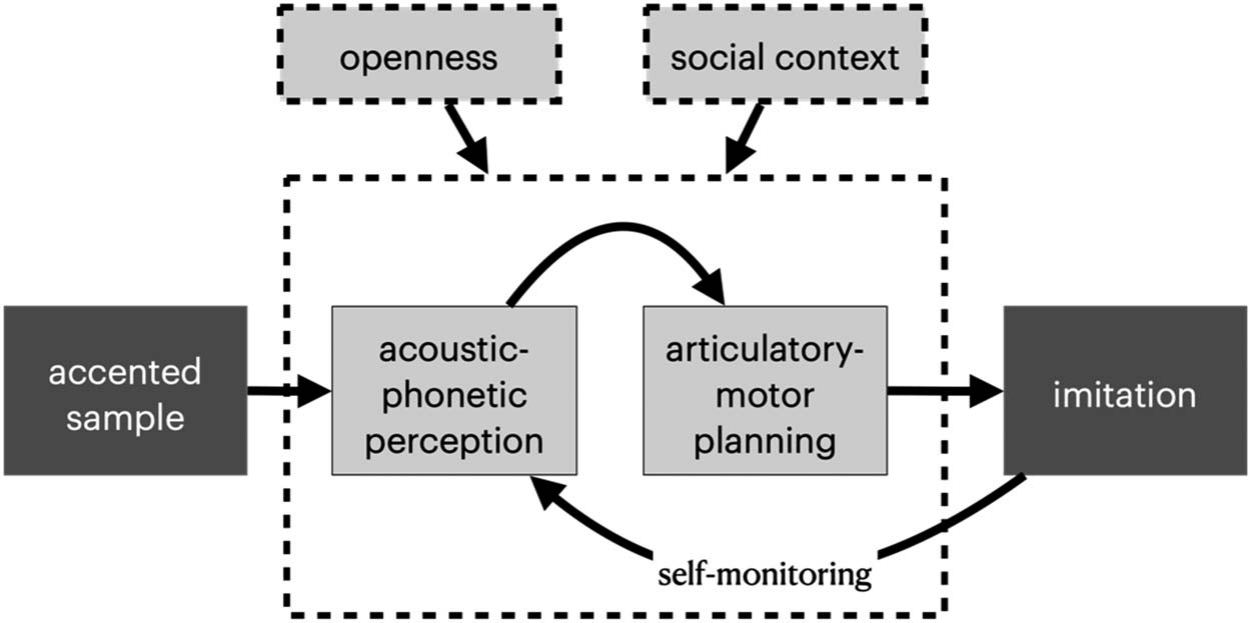
Individual differences in accent imitation ability rely on differences in perceptual and articulatory skill. We propose here that differences in trait (or state) personality, specifically openness and social context, modulate this perceptual-motor circuit.

Taken together, the set of factors identified in this study suggests that deliberate imitation skill rests on a set of core perceptual-motor skills; this is sensible since an inability to perceive accent differences or to produce them would break this perceptual-motor cycle. Yet this process is also modulated by personality and disposition to the talker. We propose that trait openness and a positive disposition to the talker essentially act as gain modulators on the perceptual-motor loop, such that imitators strengthen or weaken feedback within this loop according to their sense of social openness or alignment (see Figure 4). A strong test of this model would include intervention on perceptual or articulatory skill (i.e., training) and manipulation of state openness and disposition to the to-be-imitated talker to determine whether these factors are simply qualities of a good imitator or are actually driving the behavior. From a theoretical perspective, results suggest that accounts of imitation must not neglect low-level contributions from perceptual-motor skills, nor the role that social disposition (here measured both by general personality differences as well as specific attitudes towards the model talkers) plays in modulating, or perhaps limiting, vocal plasticity. The similarity in the predictors of individual differences in spontaneous and deliberate imitation reinforces the view that spontaneous and deliberate imitation rely on a shared set of underlying processes.

Under the assumption that our participants are making good-faith efforts to imitate speech, we argue that there may be limits dictated by individual differences in perception and production in the degree to which a talker can alter their voice. These limitations would be predicted to emerge in studies of spontaneous imitation as well, although this remains to be tested. This core ability in detection of speech differences and production of those differences can be amplified or dampened by social factors including the attitude towards the talker. Arguably, attitudes towards the talker are even more important in situations which involve direct interaction between interlocutors, which would predict that social attitudes would exert an even greater amplifying or dampening effect when real people interact.

These findings also highlight that language users are heterogeneous: individual variation in component processes that support and modulate language result in sometimes substantial differences in language perception and production (see Yu & Zellou, 2019 for helpful discussion of the implications of individual differences in phonology for theories of language architecture). Although the current study highlights differences in production, a recent report (Heffner & Myers, 2021) showed significant relationships among a set of tasks designed to tap “phonetic plasticity”—tasks that required listeners to learn new speech sounds or adapt to challenging listening conditions. Shared with the current report, vocabulary was found to be a significant predictor of perceptual phonetic plasticity. Of significant interest is the degree to which these individual differences (a) have functional outcomes for individuals (i.e., in terms of language or reading disorders), (b) participate in language change, and (c) hinge on language-specific or more domain-general processes.

### Limitations, Implications, and Future Directions

To capture global acoustic changes during the accent imitation task, we used perceptual ratings by naïve listeners as our dependent measure. These ratings had good convergence with one another, and a high correlation across different accents, suggesting that raters reliably tuned in to some aspect of the imitation. Pardo and colleagues report that perceptual ratings tend to aggregate across several acoustic cues rather than focusing on only one manipulated dimension of the signal (Pardo, 2013; Pardo et al., 2013). Further, other ways of assessing imitation success—for instance, allowing listeners to group imitation attempts in clusters, head-to-head comparison of imitation samples, or objective acoustic measures of imitation targets—might yield sensitivity to aspects of the signal that our rating data could not show. Yet of interest are exactly which aspects of the acoustic signal raters are most attuned to. In a study of phonetic accommodation, Goldinger (1998) suggested that only duration and intonation contour were reliably used by perceptual raters, although, in Pardo et al. (2013), perceptual ratings of convergence were found to be predicted by vowel spectra (a segmental-level property) as well as duration and F0. It is notable that in dialect coaching, prosodic patterns are an important target for instruction, precisely because they carry so much of one’s impression of the accent. Future work may address the acoustic correlates of these perceptual ratings, either by careful acoustic analysis of segmental properties, or by neutralizing these prominent prosodic cues—e.g., by asking raters to judge accent imitation attempts after the prosodic contour has been flattened.

Although the similarity between predictors of individual differences in spontaneous and deliberate imitation are notable, there are differences in methodology, experimental setting, and stimulus materials that preclude a direct comparison between the current study and studies of spontaneous imitation. Studies of spontaneous imitation often hone in on a particular acoustic-phonetic change (e.g., VOT, vowel space) as a marker of precise adaptation to the signal; our more general approach probing several accents, and using a general ‘imitation rating’ measure does not reach this level of precision and is instead designed to paint a picture of imitation skill with a broader brush. Many studies of spontaneous imitation involve careful manipulation of the social context (e.g., Babel, 2010; Babel et al., 2014; Pardo et al., 2012), whereas our listeners heard disembodied voices with no social context. Finally, many studies of spontaneous imitation involve convergence between speakers of the same dialect, where the acoustic-phonetic differences between talkers are likely more subtle and less salient than in the current study. Given findings from the spontaneous imitation literature suggesting that a greater difference between talker dialects promotes more convergence (Babel, 2010; Walker & Campbell-Kibler, 2015), studies using within-dialect convergence may show more subtle or hard-to-detect effects. Future studies directly comparing spontaneous and deliberate imitation abilities in more comparable settings would be needed in order to draw firm conclusions that spontaneous and deliberate imitation skills are in some sense contingent on one another.

The most important limitation of this study is that it is entirely observational. As frequently alluded to in the discussion, although predictors of accent imitation can be sensibly linked to differences in ability, confidently targeting the skills and traits that underlie accent imitation is impossible. A true test of this model requires intervention on the predictors to determine if they significantly modulate imitation success. For instance, interventions that are designed to increase trait “openness” or those that train articulatory agility or musicality will theoretically improve accent imitation. Luckily, interventions that target articulatory skill, musical ability, and notions of openness and attitude are already represented in two professional communities that train speech production, namely speech-language pathology and voice/dialect coaching for drama (e.g., Druker et al., 2019; Johnstone & Wardle, 1987; Knight, 2013; van Tellingen et al., 2023). Many of the strategies used by these professionals may already address some of these component skills (e.g., articulation drills, theater exercises that break down inhibitions, exercises for pitch variation or rhythm awareness, etc.). Future work will be needed to definitively identify the underlying components of this complex yet important skill.

## CONCLUSION

The ability to accurately imitate a voice with a different accent may seem like a niche skill. Nonetheless, imitation ability is fundamental to communication. Here we show that deliberate imitation is driven by individual differences in perceptual (musical) skill and speech-motor ability, and modulated by aspects of personality (trait openness) as well as attitudes towards the imitated speaker’s voice. These findings demonstrate that vocal imitation connects to many domains of human cognition, and situate deliberate imitation skills at an intersection between social factors, linguistic architecture, and domain-general aspects of cognition.

## ACKNOWLEDGMENTS

This paper was prepared using the ‘papaja’ package (Aust & Barth, 2020). The authors would like to thank Hannah Mechtenberg, Sahil Luthra, Pamela Fuhrmeister, Matt Phillips, David Saltzman, Anders Waldo, and Maria Murljacic for assistance with aspects of data collection and analysis.

## FUNDING INFORMATION

This work was funded by a grant from the Office of the Vice President for Research at the University of Connecticut. Myers was supported in part by an NIH grant from the National Institutes on Deafness and Other Communication Disorders (R01 DC013064, Myers, PI), and a grant from the National Science Foundation (BCS 1554810, Myers, PI) funded development of tasks used in this study.

## AUTHOR CONTRIBUTIONS

The authors made the following contributions. EBM: Conceptualization, Data curation, Formal analysis, Writing – Original draft, Writing – Review & editing; HEO: Conceptualization, Data curation, Writing – Review & editing; JS-T: Conceptualization, Writing – Review & editing.

## Note

^1^ It is important to point out that even if a quality which is thought to be fairly stable within the individual (e.g., vocabulary size, articulatory skill) predicts imitation behavior, this does not preclude the influence of other factors in modulating this behavior. Indeed, as discussed in Goldrick and Cole (2023), spontaneous imitators show non-uniformity in their adoption of acoustic-phonetic traits of the imitated voice, suggesting that there are inherent limits, arising whether from social context or linguistic structures, that place bounds on the quantity and quality of imitation.

## APPENDIX A: AFFINITY AND DIFFICULTY RATINGS

Now you will answer a few questions about the person whose voice you just heard. How much do you think the speaker’s personality conforms to the following trait? Brackets display the category of trait each prompt contributes to (see Adank, Stewart, et al., 2013; Bayard et al., 2001).

Reliable 1 (not at all) 2 3 4 5 (very much) [COMPETENCE]
Ambitious 1 (not at all) 2 3 4 5 (very much) [COMPETENCE]
Humorous 1 (not at all) 2 3 4 5 (very much) [ATTRACTIVENESS]
Authoritative 1 (not at all) 2 3 4 5 (very much) [POWER]
Competent 1 (not at all) 2 3 4 5 (very much) [COMPETENCE]
Cheerful 1 (not at all) 2 3 4 5 (very much) [ATTRACTIVENESS]
Friendly 1 (not at all) 2 3 4 5 (very much) [ATTRACTIVENESS]
Dominant 1 (not at all) 2 3 4 5 (very much) [POWER]
Intelligent 1 (not at all) 2 3 4 5 (very much) [COMPETENCE]
Assertive 1 (not at all) 2 3 4 5 (very much) [POWER]
Controlling 1 (not at all) 2 3 4 5 (very much) [POWER]
Warm 1 (not at all) 2 3 4 5 (very much) [ATTRACTIVENESS]
Hardworking 1 (not at all) 2 3 4 5 (very much) [COMPETENCE]
Powerful 1 (not at all) 2 3 4 5 (very much) [POWER]
Strong 1 (not at all) 2 3 4 5 (very much) [POWER]
Educated 1 (not at all) 2 3 4 5 (very much) [COMPETENCE]
Pleasant 1 (not at all) 2 3 4 5 (very much) [ATTRACTIVENESS]
Attractive 1 (not at all) 2 3 4 5 (very much) [ATTRACTIVENESS]
How difficult did you find it to imitate this particular accent?
Difficulty 1 (not at all) 2 3 4 5 (very)

## APPENDIX B: FULL MODEL RESULTS

**Table B1.** Full model predicting accent imitation accuracy from a set of continuous and categorical predictors.

Term	$\hat{\beta }$	95% CI	*t*	*df*	*p*
Intercept	3.53	[3.33, 3.73]	34.55	74.35	<.001
Age	−0.04	[−0.16, 0.07]	−0.77	75.69	.442
Difficulty	−0.06	[−0.08, −0.04]	−5.78	38,965.33	<.001
Power	−0.05	[−0.07, −0.02]	−4.09	37,344.61	<.001
Competence	0.06	[0.04, 0.08]	4.78	37,164.73	<.001
Attractiveness	−0.02	[−0.04, 0.00]	−1.51	39,514.47	.130
SexM	0.04	[−0.18, 0.26]	0.36	77.05	.717
BilingualMonolingual	−0.04	[−0.26, 0.17]	−0.40	76.45	.692
MusicianNonmusician	−0.11	[−0.34, 0.13]	−0.87	75.92	.385
BigFiveAgreeableness	0.03	[−0.08, 0.14]	0.57	75.89	.568
BigFiveConscientiousness	0.00	[−0.11, 0.10]	−0.04	75.73	.968
BigFiveExtraversion	−0.03	[−0.13, 0.07]	−0.55	76.06	.581
BigFiveNeuroticism	0.02	[−0.08, 0.12]	0.38	76.56	.704
BigFiveOpenness	0.10	[0.00, 0.21]	1.91	75.78	.060
Vocabulary	0.09	[−0.02, 0.20]	1.56	75.89	.122
MusicalPerception	0.10	[−0.01, 0.22]	1.76	76.44	.082
Articulation	0.13	[0.01, 0.24]	2.11	75.92	.038
PhoneticConsistency	0.05	[−0.06, 0.17]	0.89	75.66	.375
PhoneticSlope	0.00	[−0.11, 0.12]	0.08	75.75	.937
